# Supplementary material for: Molecular Characterization and Comparative Genomics of IncQ-3 Plasmids Conferring Resistance to Various Antibiotics Isolated from a Wastewater Treatment Plant in Warsaw (Poland)
Source: Antibiotics (Basel). 2020 Sep 17;9(9):613. doi: 10.3390/antibiotics9090613 (PMC7557826; doi:10.3390/antibiotics9090613)
Supplement: Supplementary file 1 [file antibiotics-09-00613-s001.pdf]

Supplementary material

Table S1. Results of in bi- and triparental mating assays of identified IncQ-3 plasmids.

		Plasmid recipients			
**Plasmid donor(s)**	**Selection (µg/mL)**	***E. coli* DH5α (Rif^{r})/*E. coli* JCB816 (Tet^{r}) (biparental)**	***E. coli* DH5α/ *E. coli* JCB816 (Tet^{r}) + *E. coli DH5a* with pRK2013 (triparental)**	***P. aeruginosa* PA01161 (Rif^{r}) (biparental)**	***P. aeruginosa* PA01161 (Rif^{r}) + *E. coli DH5a* with pRK2013 (triparental)**
Aeromonas sp. **5.4**[1]	CAZ16/RIF50	+	+	+	+
Aeromonas sp. **115**	CAZ16/RIF50	+	+	+	+
Raoultella sp. **210C**[2]	CAZ16/RIF50 TRI50/RIF50	+	+	+	+
Aeromonas sp. **426**	CIP1/TET50	-	-	ND[3]	ND
Aeromonas sp. **458**	CIP1/RIF50	-	-	-	-
Kluyvera sp. **435**	CAZ16/RIF50	-	-	-	-

Footnotes: [1]The same results also for *Aeromonas* sp. 6.45; [2]The same results also for *Raoultella* sp. 274B , *Raoultella* sp. 213C , *Raoultella* sp. 382A , *Raoultella* sp. 293, *Raoultella* sp. 385A , *Raoultella* sp. 299A , *Raoultella* sp. 376, *Raoultella* sp. 286, *Raoultella* sp. 328; [3]ND – not done

Table S2. Genes located within analysed IncQ-3 plasmids (antibiotic resistance gens are **in bold**).

Gene	Plasmid	Location	Gene length (bp)	Protein length (aa)	Predicted function
5.4_c400005	p5.4_c4	1..432	432	143	plasmid mobilization relaxosome protein MobC
5.4_c400010	p5.4_c4	416..2857	2442	813	relaxase/mobilization nuclease topoisomerase MobA/primase RepB fusion protein
5.4_c400015	p5.4_c4	2928..3791	864	287	replication protein RepA
5.4_c400020	p5.4_c4	3778..4629	852	283	replication protein RepC
5.4_c400025c	p5.4_c4	complement(4940..5803)	864	287	**class A extended-spectrum beta-lactamase GES-7**
5.4_c400030c	p5.4_c4	complement(5950..6186)	237	78	**trimethoprim-resistant dihydrofolate reductase DfrB3**
5.4_c400035	p5.4_c4	6370..7410	1041	346	class 3 integron integrase IntI3
5.4_c400040c	p5.4_c4	complement(7824..7838)	468	155	hypothetical protein
115_p200005	p115_p2	1..432	432	143	plasmid mobilization relaxosome protein MobC

115_p200010	p115_p2	416..2857	2442	813	relaxase/mobilization nuclease topoisomerase MobA/primase RepB fusion protein
115_p200015	p115_p2	2928..3791	864	287	replication protein RepA
115_p200020	p115_p2	3778..4629	852	283	replication protein RepC
115_p200025c	p115_p2	complement(4887..5441)	555	184	**AAC(6')-Ib family aminoglycoside 6'-N-acetyltransferase**
115_p200030c	p115_p2	complement(5575..6438)	864	287	**class A beta-lactamase GES-7**
115_p200035c	p115_p2	complement(6585..6821)	237	78	**trimethoprim-resistant dihydrofolate reductase DfrB3**
115_p200040	p115_p2	7005..8045	1041	346	class 3 integron integrase IntI3
115_p200045c	p115_p2	complement(8526..8993)	468	115	hypothetical protein
426_p300005	p426_p3	1..432	432	143	plasmid mobilization relaxosome protein MobC
426_p300010	p426_p3	416..2857	2442	813	relaxase/mobilization nuclease topoisomerase MobA/primase RepB fusion protein
426_p300015	p426_p3	2928..3791	864	287	replication protein RepA
426_p300020	p426_p3	3778..4659	882	293	replication protein RepC
426_p300025c	p426_p3	complement(5203..5859)	657	218	**quinolone resistance pentapeptide repeat protein QnrS2**
426_p300030c	p426_p3	complement(6697..7164)	468	155	hypothetical protein
435_c400005	p435_c4	1..387	387	128	plasmid mobilization relaxosome protein MobC
435_c400010	p435_c4	371..2797	2427	808	relaxase/mobilization nuclease topoisomerase MobA/primase RepB fusion protein
435_c400015	p435_c4	2868..3749	882	293	replication protein RepA
435_c400020	p435_c4	4048..4482	435	144	ABC transporter ATPase (frameshifted; incomplete; partial in the middle of a contig; missing start and stop)
435_c400025c	p435_c4	complement(4724..5083)	360	119	ABC transporter ATPase (frameshifted; incomplete; partial in the middle of a contig; missing start and stop)
435_c400030c	p435_c4	complement(5408..6556)	1149	382	**cephalosporin-hydrolyzing class C beta-lactamase FOX-15**
435_c400035	p435_c4	6671..7975	1305	434	IS4 family transposase
435_c400040	p435_c4	complement(8483..8950)	468	155	hypothetical protein
458_p300005	p458_p3	1..432	432	143	plasmid mobilization relaxosome protein MobC
458_p300010	p458_p3	416..2857	2442	813	relaxase/mobilization nuclease topoisomerase MobA/primase RepB fusion protein
458_p300015	p458_p3	2928..3791	864	287	replication protein RepA
458_p300020	p458_p3	3778..4659	882	293	replication protein RepC

458_p300025c	p458_p3	complement(5203..5859)	657	218	**quinolone resistance pentapeptide repeat protein QnrS2**
458_p300025c	p458_p3	complement(6586..8244)	1659	552	Aerotaxis sensor receptor protein
458_p300035c	p458_p3	complement(8715..9182)	468	155	hypothetical protein

Table S3. Bacterial strains used in this study.

Bacterial strain	Relevant characteristics	References
E. coli DH5α	Rif^R	[1]
E. coli JCB816	Tet^R	[2]
Pseudomonas aeruginosa PAO1161	Rif^R	[1]
E. coli strain DH5a pRK2013	Km^R, with pRK2013 mobilization helper plasmid	[3]
Aeromonas sp. 5.4	Caz^R, Tri^R	[4]
Aeromonas sp. 6.45	Caz^R, Tri^R	[4]
Aeromonas sp. 115	Caz^R, Tri^R	[4]
Kluyvera sp. 435	Caz^R	[5]
Aeromonas sp. 458	Cip^R	[4]
Aeromonas sp. 426	Cip^R	[4]
Raoultella sp. 210C	Caz^R, Tri^R, Mer^R	[5]
Raoultella sp. 274B	Caz^R, Tri^R, Mer^R	[5]
Raoultella sp. 213C	Caz^R, Tri^R, Mer^R	[5]
Raoultella sp. 382A	Caz^R, Tri^R, Mer^R	[5]
Raoultella sp. 293	Caz^R, Tri^R, Mer^R	[5]
Raoultella sp. 385A	Caz^R, Tri^R, Mer^R	[5]
Raoultella sp. 299A	Caz^R, Tri^R, Mer^R	[5]
Raoultella sp. 376	Caz^R, Tri^R, Mer^R	[5]
Raoultella sp. 286	Caz^R, Tri^R, Mer^R	[5]
Raoultella sp. 328	Caz^R, Tri^R, Mer^R	[5]

Footnotes: Rif – rifampicin, Tet – tetracycline, Cip – ciprofloxacin, Km – kanamycin, Caz – ceftazidime, Tri – Trimethoprim; Mer – Meropenem

Table S4. Results on Minimum Inhibitory Concentration (MICs) for donor and recipient strains for bi- and triparental mating assays.

	MIC (μg/ml)				
**Bacterial strain**	**Ceftazidime**	**Trimethoprim**	**Ciprofloxacin**	**Tetracycline**	**Rifampicin**
Aeromonas sp. **5.4**	128	>512	ND[3]	ND	1
Aeromonas sp. **6.45**	>128	>512	ND	ND	2
Aeromonas sp. **115**	>128	>512	ND	ND	4
Raoultella sp. **210C**[1]	>128	>512	ND	ND	16
Raoultella sp. **382A**[2]	>128	>512	ND	ND	32

Aeromonas sp. **426**	ND	ND	4	1	128
Aeromonas sp. **458**	ND	ND	2	1	1
Kluyvera sp. **435**	128	ND	ND	ND	2
E. coli DH5α (Rif$^+$)	0,125	0,5	<1	1	>256
E. coli JCB816 (Tet$^+$)	4	<0,5	<1	128	8
Pseudomonas aeruginosa PAO1161 (Rif$^+$)	2	128	<1	8	>256

Footnotes: [1]The same MIC results also for *Raoultella* sp. 274B , *Raoultella* sp. 213C , *Raoultella* sp. 385A , *Raoultella* sp. 299A , *Raoultella* sp. 376, *Raoultella* sp. 286, *Raoultella* sp. 328; [2]The same MIC results also for *Raoultella* sp. 293; [3]NP – not done

Table S5. Primers used in this study.

**Primer name**	**Nucleotide sequence**	**References**
MultiGES_for	AGTCGGCTAGACCGGAAAG	[6]
MultiGES_rev	TTTGTCCGTGCTCAGGAT	[6]
FOXMF	AACATGGGGTATCAGGGAGATG	[7]
FOXMR	CAAAGCGCGTAACCGGATTGG	[7]
qnrS_for	GCAAGTTCATTGAACAGGGT	[8]
qnrS_rev	TCTAAACCGTGAGTTCGGCG	[8]

References:

1. Bartosik, A.A.; Glabski, K.; Jecz, P.; Mikulska, S.; Fogtman, A.; Koblowska, M.; Jagura-Burdzy, G. Transcriptional Profiling of ParA and ParB Mutants in Actively Dividing Cells of an Opportunistic Human Pathogen Pseudomonas aeruginosa. *PLoS One* **2014**, *9*, doi:10.1371/journal.pone.0087276.
2. Bardwell, J.C.A.; McGovern, K.; Beckwith, J. Identification of a protein required for disulfide bond formation in vivo. *Cell* **1991**, *67*, 581–589, doi:10.1016/0092-8674(91)90532-4.
3. Figurski, D.H.; Helinski, D.R. Replication of an origin-containing derivative of plasmid RK2 dependent on a plasmid function provided in trans. *Proc. Natl. Acad. Sci. U.S.A.* **1979**, *76*, 1648–1652, doi:10.1073/pnas.76.4.1648.
4. Piotrowska, M.; Przygodzińska, D.; Matyjewicz, K.; Popowska, M. Occurrence and Variety of β-Lactamase Genes among Aeromonas spp. Isolated from Urban Wastewater Treatment Plant. *Front Microbiol* **2017**, *8*, 863, doi:10.3389/fmicb.2017.00863.
5. Piotrowska, M.; Kowalska, S.; Popowska, M. Diversity of β-lactam resistance genes in gram-negative rods isolated from a municipal wastewater treatment plant. *Ann Microbiol* **2019**, doi:10.1007/s13213-019-01450-1.
6. Dallenne, C.; Da Costa, A.; Decre, D.; Favier, C.; Arlet, G. Development of a set of multiplex PCR assays for the detection of genes encoding important -lactamases in Enterobacteriaceae. *Journal of Antimicrobial Chemotherapy* **2010**, *65*, 490–495, doi:10.1093/jac/dkp498.
7. Perez-Perez, F.J.; Hanson, N.D. Detection of Plasmid-Mediated AmpC -Lactamase Genes in Clinical Isolates by Using Multiplex PCR. *Journal of Clinical Microbiology* **2002**, *40*, 2153–2162, doi:10.1128/JCM.40.6.2153-2162.2002.
8. Kim, H.B.; Park, C.H.; Kim, C.J.; Kim, E.-C.; Jacoby, G.A.; Hooper, D.C. Prevalence of Plasmid-Mediated Quinolone Resistance Determinants over a 9-Year Period. *Antimicrob Agents Chemother* **2009**, *53*, 639–645, doi:10.1128/AAC.01051-08.
